# Complete Genome Sequence of *Serratia rubidaea* Isolated in China

**DOI:** 10.1128/genomeA.00283-16

**Published:** 2016-04-28

**Authors:** Xue Yao, Qiang Sun, Wenli Liu, Xiuyun Yin, Guangqian Pei, Yunfei Wang, Xiaoping An, Zhiqiang Mi, Yaping Luo, Yigang Tong, Shuiping Chen

**Affiliations:** a School of Criminal Science and Technology, People’s Public Security University of China, Beijing, China; b State Key Laboratory of Pathogen and Biosecurity, Beijing, China; c 307th Hospital of PLA, Beijing, China

## Abstract

We report here the first complete genome of *Serratia rubidaea*, isolated from a patient in China.

## GENOME ANNOUNCEMENT

*Serratia rubidaea* mainly exists in soil, water, and food, and it is usually nonpathogenic. But when a patient's immunity is decreased, it can cause invasive infection, such as pulmonary infection and traumatic infection (1, 2). We report here the first complete genome sequence of *Serratia rubidaea*, isolated from a patient in China.

The male patient was 55 years old and was admitted to the hospital due to aphasia and right hemiparesis. Then he was diagnosed with left basal ganglia hemorrhage, accompanied by pulmonary infection. Intracerebral hematoma drilling and drainage were carried out on the first and fourth days after admission. *Klebsiella pneumonia* was isolated from the sputum samples 2 days after admission. And *Serratia rubidaea* was isolated from the sputum samples 4 days after admission.

DNA of *Serratia rubidaea* was extracted with a ChargeSwitch gDNA mini bacteria kit (Thermo Fisher Scientific, USA)*.* Whole-genome sequencing was carried out using an Ion Torrent PGM sequencer with a 400-bp single-end library and MiSeq PE250 sequencer with a 5-kb mate-pair library. The Ion Torrent PGM sequencer generated 1,325,318 reads (approximately 69× coverage of the genome), and the MiSeq PE250 sequencer generated 1,428,964 pair of reads (approximately 139× coverage of the genome). Then, sequences were *de novo* assembled through the Newbler assembler version 2.9. The complete genome of *Serratia rubidaea* contains a circular 4,922,834-bp chromosome, with a 59.20% G+C content and no plasmids.

The chromosome was annotated through the Rapid Annotations using Subsystems Technology (RAST) server (3). A total of 4,486 coding DNA sequences were predicted, and 654 of them are hypothetical proteins. Ninety-six RNA genes were detected on the chromosome of *Serratia rubidaea*, and 74 of them are tRNA genes; others are rRNA operons. All of the predicted coding DNA sequences were blasted through the Drug Resistance Genes Database and the Virulence Genes Database constructed by our lab: 60 drug-resistance-related genes were found, including *rpoB*, *mdtB*, *mdtC*, *parC*, *acrB*, *katG*, and *cpxA*; and 428 virulence-related genes were found, including *mrsA*, *htpB*, *hemE*, *tuf*, *pvdI*, *stfC*, and *rfaD*.

There is only one draft genome of *Serratia rubidaea* in GenBank so far (4), and we hope that our sequence will be helpful for genomic comparisons and related case studies of the *Serratia* genus.

### Nucleotide sequence accession number.

The complete genome sequence of *Serratia rubidaea* has been deposited in NCBI GenBank under the accession number CP014474.
